# Clinical Lecture on the Study of Children’s Diseases

**Published:** 1866-03

**Authors:** Charles West

**Affiliations:** Physician to the Hospital


### CLINICAL LECTURE ON THE STUDY OF CHILDREN'S DISEASES.

Given in the Hospital for Sick Children. By CHARLES WEST, M.D., Physician to the Hospital.

GENTLEMEN:—A very wise and good man, to whom I owe much of whatever I have learned of my profession (Dr. Latham) makes, somewhere, the remark, that he was struck at the outset of his career with how, in a large hospital, knowledge was continually running to waste for the want of some one to gather it. He says, too, that this which struck him then, struck him even more forcibly in after years; the old experience, in short, "*Ars longa, vita brevis,*" which is realized more and more as the shadows lengthen and the day goes down.

I feel it specially with reference to this hospital, because here, or in inquiries such as its wards suggest, my time, and thoughts, and energies have been engaged for the past twenty-five years, and I rejoice to see you here to-day, gentlemen, because in some of you I trust that I may find fellow-laborers —men already schooled by previous study, and who will be able as well as ready to gather, for the common benefit, some of that knowledge which will otherwise be but too likely lost.

But I am glad, also, to see others here, who as yet are but imperfectly trained, because, while they have much to learn, they have come here, to one of the best places in which to learn it, since disease may here be studied in its simplest forms.

It has been recommended by some, most fitted to give advice, that the student of medicine should begin with the diseases of the eye, since, through its transparent coats, as through a glass, the various processes of disease and recovery may be seen transacted, and "many of the little wonderful details in the nature of morbid processes may be learned, which, but for the observation of them in the eye, would not have known them at all." The ophthalmic wards of a hospital must, indeed, be revisited at a later period for the sake of the special knowledge to be obtained there, but they may well be visited at first for the elementary teaching which they afford.

Somewhat in the same way, you may come at two periods of your career to the study of children's diseases. *First*, to observe disease in its simplest conditions; then *later*, to investigate the peculiarities of symptoms which result from the tender age of your patients, and the modifications of treatment, which, on that account, may be required.

First, I said, to study disease in its simplest form. The chemist who analyzes a substance, submits it to various processes in order to remove from it all extraneous matters, and then applies to it tests to determine its real nature. This which the chemist does, however, is very difficult indeed, in the investigation of disease. Pure pathology is the doctrine of disease, unmodified by the intervention of disturbing causes from without or from within. To this in adult age we scarcely ever attain. The body, even in apparent health, yet tends imperceptibly to decay. We study disease in its influence on parts already damaged. The follies of youth, the vices of maturer age, the anxieties of business, the failure of hope, all leave their impress on the body, diminish its reparative powers, and render the different organs inapt to do their duty; so that almost all disease appears in a complicated, scarcely ever in a simple, form.

Care, too, which sits at the bed's head of the grown person, does much to retard recovery and to complicate disease. "Is your mind at ease?" said his physician to poor Goldsmith on his death-bed, observing how his pulse outbeat the frequency for which his bodily ailment would have accounted. In childhood there is little or none of this; no regret for the past; no dread of the future. The present is the world in which little children live; pain past is almost forgotten; and this mental tranquility contributes in no small degree to their recovery.

But I will no longer occupy you with insisting on things with which a little time spent here will make you quite familiar. I

will rather make a hasty survey of some of the cases which are now in the hospital, or which have been here so recently that many of you have had the opportunity of seeing them. I select them very much for the illustration they furnish of the unsolved problems which I want some of you to try to answer.

A little boy, aged 19 months, was admitted into the hospital, on the fourth day of an attack of pneumonia of both lungs. His respirations were 60; his pulse beat 148 in the minute. There was dulness at the base of both lungs, especially of the right; fine crepitation was heard below both scapulæ. I scarcely need add that the child seemed very ill. He was drowsy, but at the same time restless. He was very hot and his skin dry. He had some cough, but not very much. A mustard poultice was applied to the back and the chest. A little ammonia was given with small doses of ipecacuanha; beef-tea and wine for food.

In the night, the distress and restlessness were extreme until relieved by spontaneous vomiting; but on the afternoon of the fifth day the child was already better; the respiration had fallen in frequency to 44; and there was slightly improved resonance of the chest. Improvement continued. On the ninth day the respiration had fallen to 21, and the pulse to 124; percussion yielded an almost natural sound; and some largish crepitation was the only evidence remaining of the dangerous illness.

Now, here the recovery took place speedily and decisively, and in a way in which one could not refer it to the remedies employed. Nor is this a solitary case; it is one of many to which attention has of late years been especially called, which raise the question as to when and how far an expectant treatment may be adopted in inflammation of the lungs. It suggests to you the importance of determining the period of pneumonia at which spontaneous improvement is most likely to occur, the circumstances which in any given case justify you in expecting it, and those which, on the other hand, render its occurrence doubtful. Further, there remains the important question whether, though recovery would take place independent of treatment, it yet occurs sooner, or is more complete if treatment is adopted than if the case is let alone.

A girl, 7¼ years old, was admitted with the following history: Nine months before, she suffered from severe pain in her limbs, which yet did not constantly keep her in bed; but she was up every morning, and then as afternoon came on, grew worse, and went to bed. During much of this time her heart beat very much, and at the end of a month, when the pains in her limbs had already ceased, she suffered so much from her heart that she was confined to her bed for six weeks. When better, she attended for some months as an out-patient, but six months after her illness began, her legs swelled, and her breath became short, and at length she came for admission here.

The heart's impulse was visible in the fourth, fifth, and sixth interspaces; the apex beat in the sixth interspace, one and a-half inch outside the nipple line. The upper dulness limit reached to the third rib, and the inner to a finger's breadth to right of the sternum. The oblique diameter of the heart was five and three-quarter inches, the transverse five, the longitudinal three and three-quarter inches; while, as you can now see for yourselves, there is a very manifest bulging of the whole heart's region. There was a prolonged wheezing systolic murmur heard at the apex, which diminished rapidly in loudness towards the base; the second sound was inaudible at the apex, but clearly heard at the base of the heart.

I am not going to trace this child's history in detail. She got relief from treatment, went out much better in three months, but soon came back in a state of great distress, for now pericarditis had come on. For some time she seemed likely to die, but once more got better, and you see her now, eighteen months after the rheumatism in which her sufferings began.

Now, here you have a case of heart disease, with enormous dilatation of the organ, succeeding to a comparatively slight attack of rheumatism. Each year adds to the child's sufferings, from which she will find rest only in an early grave.

Why is this so? Why does even a very small amount of valvular disease tend in some instances to produce a large amount of dilatation?

It is not an invariable occurrence. So little, indeed, is it invariable, that Dr. Latham notices the probable existence of some compensating power in the young heart, by which atonement is made for the effects of valvular disease: "a certain *protective* power possibly inherent in the growing heart, whereby it can accommodate its form and manner of increase to material accidents, and so suppress or counteract their evil tendencies."

But why is this sometimes? Why not always? Why not often? Is this happy issue rarer now than formerly, and if so, can it be that the change in practice which recent years have brought with them—the abolition of depletion, the disuse of mercury, have rendered the cure of rheumatic affections of the heart less complete than formerly? Or, is it only, and this I

apprehend to be the case, that our diagnostic skill and pathological knowledge have outstripped by far our therapeutical resources, that we discover the ills which we are impotent to cure?

A strong-looking, well-made girl, 10 years and 9 months old, began to suffer causelessly from chorea, three weeks before her admission into the hospital. There was no history of rheumatism in her family, nor had she herself presented any rheumatic symptoms, though there was a weak systolic bruit audible at the apex of her heart, which persisted, but did not increase, either in extent or loudness, during the whole of her illness. the choreic movements were at first limited to the left arm, but they increased rapidly, in spite of treatment, so that a month after admission the child was compelled to be placed in a bed padded all round, on account of the violence of her movements, while deglutition was very imperfect, and speech almost abolished.

She remained in ths hospital for three months, at the end of which time she was almost well, and was sent into the country. She was submitted to very varied treatment, but without benefit, and her eventual improvement was spontaneous. For a time she improved in the country, but at the end of two months returned with a relapse of all her former symptoms, though their severity was far less than on the former occasion. In this instance, medicine seemed just as unavailing as before. The child began spontaneously to improve at the end of one month, was well at the end of two months, and has, I believe, since continued so, though the time is yet too short to feel sure of the permanence of her recovery.

Here, again, are several questions which await an answer. Why is the first attack of chorea almost always the most severe? Why is there no definite relation between the severity of chorea and the severity of the heart affection, and why is the heart sometimes quite unaffected, even though the chorea is very severe? Lastly, why is the heart affected at all, since the assumption of its rheumatic character, though true to a certain extent, is yet by no means always tenable?

Further, what clear indications can be laid down for the treatment of chorea besides the two furnished by the existence of constipation on the one hand, and anæmia or debility on the other, and the combined use of purgatives and tonics which they suggest?

Zinc and antimony, strychnine and belladonna, shampooing and sulphur baths have all been used in the treatment of chorea.

When is the one right, when the other, or in what combinations are they best employed? In what combinations? for I would not have you fall into the error into which the prevalent folly of homœopathy may imperceptibly lead you, of supposing that in order to act at all each remedy must be employed alone. He is the best physician, who knows best not only what remedies to use, but in what combinations; as the skilful general trusts not to his infantry alone, nor alone to his cavalry, but gains his victory most surely and most quickly by using different troops in combination; or,

> "As many arrows loosed several ways
> Fly to one mark."

Two more cases, and I have done for to-day. One is in the hospital now, the other recently left.

A boy, 8¼ years old, was always a backward child, and while teething had three attacks of convulsions. Three months since he seemed causelessly languid for a fortnight; and then he was suddenly seized with vomiting. For six weeks together the vomiting returned daily or every other day. It was associated with an increased languor, and, by degrees, with drowsiness, and with pain in the occiput, which, though constant, became sometimes so severe as to make him scream aloud. A month after the commencement of these symptoms he was first observed to squint, and at the end of two months he had a fit which lasted for half an hour. In the ensuing month these fits returned six times. The vomiting ceased after the first fit, but the other symptoms continued, and became associated with pain on any movement of the limbs, and three weeks before his admission into the hospital it was first observed that his pupils were dilated, and that he had lost the power of sight.

He was a pale, thin child, with a peculiarly wretched expression of countenance; absolutely blind—his right eye looked straight forwards, his left inwards, and both were in a state of constant motion. He had complete power over his limbs. His headache was not constant; his appetite was good, and he did not vomit during the fortnight that he remained in the hospital. Nothing, howeqer, seemed for a moment to amuse or please him, and he was allowed to go home, all the more readily, that his case was not one which held out much prospect of benefit from treatment.

What was this case? There was no family history of tubercle, nor did the boy present any appearance of it. Still the symptoms are not those of any acute inflammatory disease, and I should be disposed to imagine that they were due to the gradual development of some tumor (and these tumors are almost always tuberculous,) which, arising at the base of the brain, had, by degrees, increased until by pressure on the optic nerve it had abolished the power of vision. And here it may remain stationary, though more probably it will continue to grow until it causes death, either suddenly by some outpouring of blood from the vessels at the base of the brain, or more slowly, by the production of inflammation, or effusion, into the ventricles consequent on pressure on the veins of Galen.

Here is, lastly, another case, somewhat obscure, indeed, but yet less so, I take it, than the preceding one:—

A girl, 8½ years old, whose father and two of her brothers had died with symptoms of brain disease, had suffered for a fortnight from troublesome cough, when she seemed unusually heavy, was attacked by violent sickness with headache, and sank speedily into a state of stupor, which continued with intervals, during which her mind wandered and she rambled in her talk, for 36 hours. At the end of this time consciousness returned, and the child sat up in bed, and showed some gleams of cheerfulness, but the pulse, which had been irregular during the state of stupor, still continued so, and the head was held somewhat retracted. Pain in the head and some retraction of it continued, though the child was well enough to be up, and moved about the ward.

She left by her mother's wish in a fortnight, and at the end of another fortnight she returned, much emaciated, complaining of pain which was now referred more to the ears than to the head, of pain also in the neck and in the right shoulder, towards which her head was inclined.

On this occasion she remained in the hospital four weeks. During this time she grew thinner and thinner, her skin became harsh, her abdomen retracted and tender to the touch, and her head was still drawn back as before. Auscultation now found the breathing weak everywhere, but especially so at the apex of the left lung, and percussion there was obviously dull. There was no vomiting, however. The bowels, once constipated, had now become regular, the complaints of headache were less constant, and the pulse had lost its irregularity. General tuberculosis was advancing; the mischief in the brain, I suppose, was stationary.

What is the import of this sudden development of the signs of cerebral disease, and what of their spontaneous passing into abeyance? If we could learn to answer these inquiries aright, we might possibly do something to arrest disease, even though we were unable to effect its cure. Here, then, is another problem which I leave for your consideration.

But, say you, you came here to be told what I do know, and I have talked to you almost entirely of what I do not know, and that is not the object of a lecture, the purpose of which should be to impart positive knowledge.

Gentlemen, it is not quite so. The acquisition of knowledge implies an active, not a passive, state, and to this it was my object to excite you. It is when you seek, as for riches, and search, as for hid treasure, that you gain it; so, at least, said the man wiser than other men, and who himself wrote of all things, from "the cedar of Lebanon, to the hyssop that groweth on the wall."

You have come to the study of medicine, furnished far differently from those who, like myself, entered on it more than 30 years ago. It is but right that you should turn these advantages to good use. We are, indeed, as has been well said, like people standing together on a hill, which I have climbed before you, and I, to whom the landscape is in some measure familiar, may say to you look here and look there, and you will see this and that. But further, I say to you, the objects there are indistinct to me; but you have perspective glasses of higher power than mine; turn them in this direction or in that, and you may, with patience, discern clearly what I can see but partially, or, with my imperfect instrument, perhaps cannot see at all.

If you visit the wards of this hospital, I may, too, do some of you the service, that I may point out to you what is worth the seeing, and may help to guard you from the dangers of the young student—that of playing with the instrument itself, vain, perhaps, of his dexterity in its use, or of turning it thoughtlessly on trifling things not worth the investigation.

You must not forget that it is your duty, as it is mine, to map out the country for the use of yourselves and of future travellers, to seize its great features, which may serve as landmarks, and not to waste your time on some quaint tree or curious rock which lies quite out of the path along which you have to journey. "*Nisi utile est quod agimus vana est gloria nostra*," should be your motto, though in a different and lower sense, indeed, from that in which it was employed by the inspired penman some eighteen hundred years ago.—*Med. Times & Gaz.*, Oct. 28th, 1865, *and Phila. Medical News.*

